# Trends and Variations in the Rates of Hospital Complications, Failure-to-Rescue and 30-Day Mortality in Surgical Patients in New South Wales, Australia, 2002-2009

**DOI:** 10.1371/journal.pone.0096164

**Published:** 2014-05-01

**Authors:** Lixin Ou, Jack Chen, Hassan Assareh, Stephanie J. Hollis, Ken Hillman, Arthas Flabouris

**Affiliations:** 1 Simpson Centre for Health Services Research, South Western Sydney Clinical School & Australian Institute of Health Innovation, University of New South Wales, Sydney, New South Wales, Australia; 2 Intensive Care Unit, Royal Adelaide Hospital, Adelaide, South Australia, Australia, Faculty of Health Sciences, School of Medicine, University of Adelaide, Adelaide, Australia; Johns Hopkins Bloomberg School of Public Health, United States of America

## Abstract

**Background:**

Despite the increased acceptance of failure-to-rescue (FTR) as an important patient safety indicator (defined as the percentage of deaths among surgical patients with treatable complications), there has not been any large epidemiological study reporting FTR in an Australian setting nor any evaluation on its suitability as a performance indicator.

**Methods:**

We conducted a population-based study on elective surgical patients from 82 public acute hospitals in New South Wales, Australia between 2002 and 2009, exploring the trends and variations in rates of hospital complications, FTR and 30-day mortality. We used Poisson regression models to derive relative risk ratios (RRs) after adjusting for a range of patient and hospital characteristics.

**Results:**

The average rates of complications, FTR and 30-day mortality were 13.8 per 1000 admissions, 14.1% and 6.1 per 1000 admission, respectively. The rates of complications and 30-day mortality were stable throughout the study period however there was a significant decrease in FTR rate after 2006, coinciding with the establishment of national and state-level peak patient safety agencies. There were marked variations in the three rates within the top 20% of hospitals (best) and bottom 20% of hospitals (worst) for each of the four peer-hospital groups. The group comprising the largest volume hospitals (principal referral/teaching hospitals) had a significantly higher rate of FTR in comparison to the other three groups of smaller-sized peer hospital groups (RR = 0.78, 0.57, and 0.61, respectively). Adjusted rates of complications, FTR and 30-day mortality varied widely for individual surgical procedures between the best and worst quintile hospitals within the principal referral hospital group.

**Conclusions:**

The decrease in FTR rate over the study period appears to be associated with a wide range of patient safety programs. The marked variations in the three rates between- and within- peer hospital groups highlight the potential for further quality improvement intervention opportunities.

## Introduction

The concept of failure-to-rescue (FTR) was first coined by Silber and colleagues in 1992 [1] with the intention of measuring potentially preventable deaths after surgical complications. The concept has been used in the United States (US) as a nursing sensitive indicator by the National Quality Forum [2] and a patient safety indicator (PSI) by the Agency for Healthcare Research and Quality (AHRQ) [3]. While the original concept of FTR covered a wide range of surgical complications, the AHRQ definition focused on surgical patients who developed at least one of six complications during hospitalisation as one of its PSIs: acute renal failure, deep vein thrombosis (and/or pulmonary embolism), pneumonia, sepsis, shock (and/or cardiac arrest), and gastrointestinal bleeding (and/or ulcer) [3]. Given the growing recognition of rapid response systems (RRS) for the timely identification and response to in-hospital deteriorating patients, the term of FTR is also used for evaluating the effectiveness of RRSs [4], [5]. In this context, the measure of FTR can estimate the entire organization’s ability to prevent avoidable complications such as unexpected cardiac arrest and related deaths for all hospital patients, not just surgical patients [3], [6]–[8]. Over the past decade, FTR has been widely used as one of seventeen patient safety indicators developed by AHRQ for quality measurement and hospital comparison purposes [9]–[14]. Failure to achieve such targets also carries financial consequences [15]. In the US, the incidence of FTR was the most common PSI accounting for 16.9% of the total number of hospital incidents [10].

Despite the widespread recognition of problems occurring in patient safety, and the plethora of quality improvement initiatives implemented, the progress in patient safety improvement remains slow [16]–[18]. There is still a need for universally accepted patient safety indicators for evaluating quality improvement programs, reporting health system performance and building a self-learning health-care system [16], [17]. In contrast to the US, however, there is no published research or formal reporting of FTR in Australia, or attempts to correlate FTR with other estimates of patient safety.

Thus, the aims of this study were to 1) explore trends in the rates of complications, FTR and 30-day mortality in acute public hospitals in New South Wales (NSW), Australia, between 2002–2009; and, 2) estimate the variations in such rates among all NSW hospitals and between the bottom 20% of hospitals (worst outcomes) and top 20% of hospitals (best outcomes) by peer hospital groups.

## Methods

### Data Source

We conducted this study using data from the NSW Admitted Patient Data Collection (APDC). The APDC is administrated by the NSW Ministry of Health as a census of all admitted patient services provided by NSW public and private healthcare facilities. The APDC includes information on patient demographics, medical conditions and procedures, hospital characteristics, and separations (discharges, transfers and deaths) from all public and private hospitals, as well as day procedure centres. The medical records for each episode of care in the APDC were assigned with codes based on the International Statistical Classification of Diseases and Related Health Problems, Tenth Revision, Australian Modification (ICD-10-AM) [19]. NSW has implemented the ICD-10-AM since 1998 and each public hospital has certified and trained coders following standardised procedures to extract the information from medical records. Surgical patient records for any of the six AHRQ-defined FTR complications (acute renal failure, deep vein thrombosis [and/or pulmonary embolism], pneumonia, sepsis, shock [and/or cardiac arrest], and gastrointestinal bleeding [and/or ulcer]) were subsequently identified.

### Study Population

**Figure 1:**
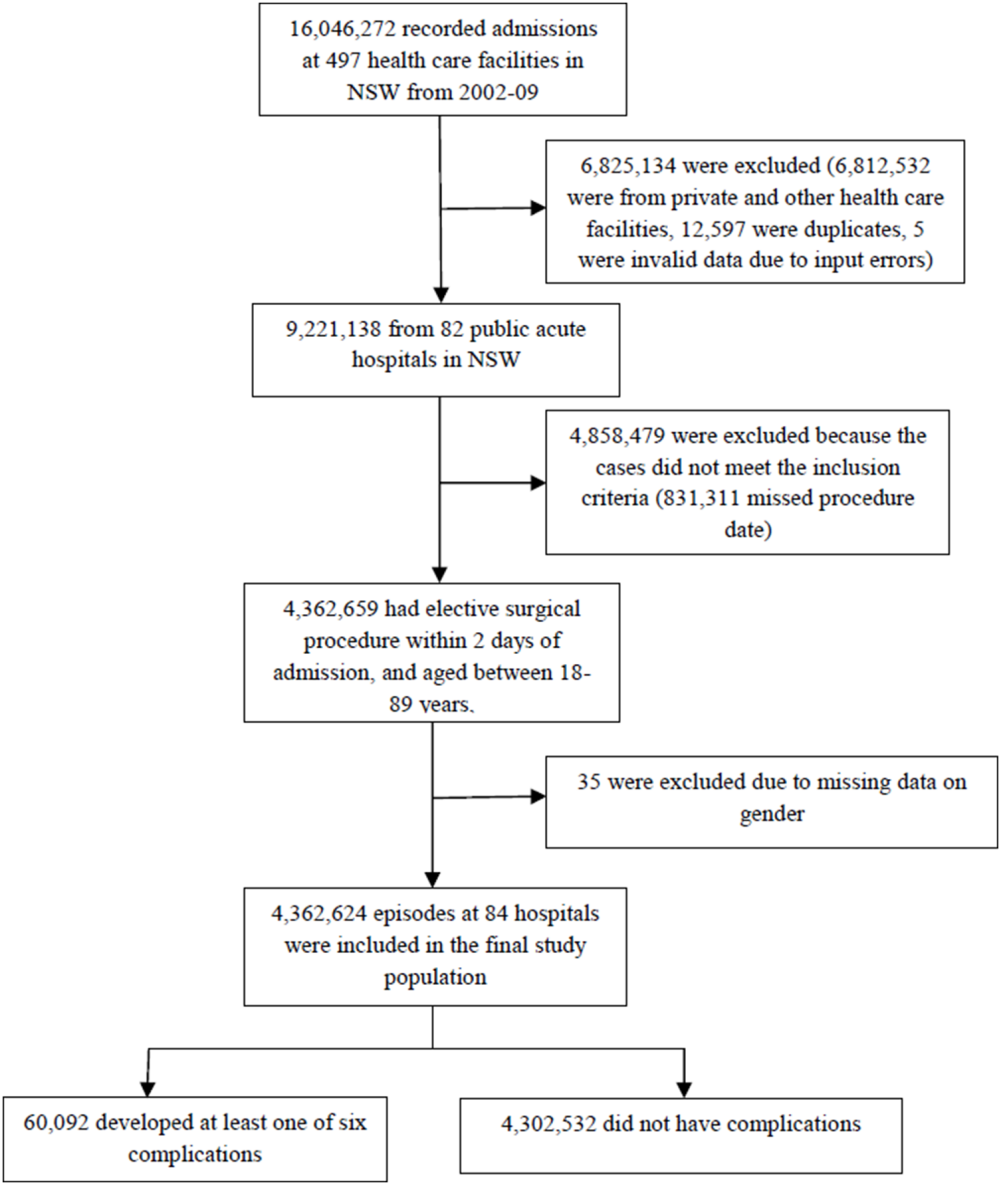
Flow chart illustrating the derivation of the study population.

Our study included the data from 82 public acute care hospitals in NSW between 1^st^ January 2002 and 31^st^ December 2009, excluding two children’s hospitals. We defined the study population by using the inclusion criteria for FTR developed by the AHRQ [3]. Elective surgical patients aged 18 years or over were identified through the source of admission code, procedure code and procedure date for an operation. Patients who had a principal procedure within two days of admission were included in the study. We excluded those who were 90 years or older, those who were transferred to an acute hospital, and those with missing data in discharge status, gender, age, year, or principal diagnosis. The selected data from the APDC were linked to the NSW Registry of Births, Deaths and Marriages data, which includes all death records in NSW, in order to identify those patients who died after discharge but within 30 days of admission. The way in which the study population was derived is presented in Figure 1. This study was approved by the NSW Population & Health Services Research Ethics Committee (LNR/11/CIPHS/64).

### Study Outcomes

Three study outcomes were measured as rates of:

**1.** In-hospital surgical complications: defined as all surgical admission patients who developed at least one of the six complications as per AHRQ definition. We then translated the ICD-9-CM based AHRQ definition using the ICD-10-AM codes developed by the Victorian Health Department [20]. The six complications were based on 54 secondary diagnostic codes in the APDC;
**2.** Failure to Rescue: defined as deaths during hospitalisation in surgical patients who developed at least one of the six complications as listed above;
**3.** 30-day mortality: defined as deaths within 30 days of all surgical admissions, including deaths after hospital discharge.

We used 30-day mortality instead of in-hospital mortality as the outcome measure given the report that 30-day mortality could remove the bias introduced by in-hospital mortality in hospitals with a shorter length of stay [21]. Both 30-day hospital mortality and in-hospital complication rates were presented as incidence per 1,000 admissions within each year between 2002 and 2009, inclusively.

### Patient Demographic and Hospital Characteristics

Patient demographic information included age, gender, country of birth, marital status, major principal diagnostic diseases, and advantage and disadvantage index scores of Socio-Economic Indices For Areas (SEIFA) [22]. We categorised the SEIFA scores into four classes (1^st^ quartile  =  most disadvantaged areas and 4^th^ quartile  =  most advantaged areas) representing patient socio-economic position. Principal diagnostic diseases included ten conditions which were the most common diseases or serious conditions as defined by Iezzoni and colleagues [23]. These ten conditions were identified through principal diagnostic coding (ICD-10-AM) at admission using the methodology developed by Quan et al.[24].

Hospital characteristics included the local health district (metropolitan, rural and regional NSW), peer group (A1: principal referral, usually teaching hospitals; A3: ungrouped acute; B: major metropolitan and non-metropolitan; C1: district group 1; and, C2: district group 2), and major procedure group. Peer hospital groups are divided into those of similar type and size, ranging from treating 25,000 or more acute case-mix weighted separations per annum in the principal referral group through to treating 2,000 or more (but less than 5,000) acute case-mix weighted separations per annum in district group 2 [25]. Using appropriate procedure codes from ICD-10-AM (Appendix S1), we defined six groups of major surgical procedures including coronary-artery bypass graft (CABG), abdominal aortic aneurysm (AAA) repair, total hip replacement, total knee replacement, cholecystectomy, and other surgical procedures.

### Statistical Analysis

We used linear regression to test mean differences between the years for continuous variables, and multinomial logistic regression for categorical variables. The interaction effect was tested between years and some of the characteristic variables such as age group, gender, local health district, and peer hospital group. If any test was statistically significant, we then stratified the results by either the characteristic variable or the calendar year of admission. Poisson regression models were performed to directly evaluate adjusted risk ratios (RR) for outcome variables using the calendar years as indicator variables, with 2002 as the reference year. We also examined quadratic time trends of the outcomes in the bivariate and adjusted analysis, using calendar year as a continuous variable. The risk factors in the model were patient demographic and hospital characteristics. We examined the Elixhauser and the Charlson Index comorbidities based on the ICD-10 coding scheme in the preliminary analyses [24]. We did not include the Elixhauser and Charlson Index score in the adjusted analysis given the recent report on the potential bias introduced by these methods [26]–[28]. Hospitals were ranked by the quintile of complication, FTR or 30-day mortality outcomes, and we compared the three outcomes between the best and worst 20% hospitals within a specific peer hospital group. We also examined variations in all three outcomes for the six groups of surgical procedures within the principal referral (or teaching hospital) group because most of the major surgical procedures were performed in teaching hospitals.

We used the Huber/White/Sandwich estimator in all statistical analyses to account for hospital clustering effect [29]. Statistical significance was calculated with 95% confidence intervals (CIs) and all analyses were performed using STATA 12 (StataCorp. College Station, TX).

## Results

### Hospital and Patient Characteristics Over the Study Period

A total of 4,362,624 elective surgical admissions between 2002 and 2009 were included in the analysis (Table 1). Gender was split evenly (P = 0.15). The average age between 2002 and 2009 increased from 55.9 to 58.2 years old (P<0.001). Over the study period, there was an increase in the average age of: those in the oldest age group (>75 years) (P<0.001); Asia-born patients (P = 0.01); patients living in disadvantaged areas (lower SEIFA score); and, patients admitted for: congestive heart failure (P<0.001), anaemia (P = 0.01), and chronic pulmonary disease (P = 0.05).

**Table 1:** Patient and hospital characteristics.

Characteristics	Year groups								
	2002–2003		2004–2005		2006–2007		2008–2009		P-value for trend
	n %		n %		n %		n %		
**Age**									
> = 18yr&<35yr	164039	18.1	185840	18.5	204504	17.4	204800	16.1	Ref
> = 35yr&<55yr	228480	25.2	244385	24.3	275973	23.4	300731	23.7	0.33
> = 55yr&<75yr	346783	38.3	375105	37.2	446357	37.9	479731	37.7	0.16
> = 75yr<90	166128	18.3	201916	20.0	251749	21.4	286103	22.5	<0.001**
Mean±SD	55.9±0.02		56.3±0.03		57.5±0.03		58.2±0.03		
**Sex**									
male	409335	45.2	454785	45.2	549310	46.6	600569	47.2	0.15
female	496095	54.8	552461	54.8	629273	53.4	670796	52.8	Ref
**Marital status**									
married	533495	58.9	595291	59.1	693837	58.9	750333	59.0	Ref
single	346023	38.2	384487	38.2	460070	39.0	501112	39.4	0.29
unknown	25912	2.9	27468	2.7	24676	2.1	19920	1.6	0.07
**Country of birth**									
Australia and New Zealand	606467	67.0	670600	66.6	789788	67.0	831038	65.4	Ref
UK, US & Canada	51750	5.7	56494	5.6	65306	5.5	70744	5.6	0.92
Non-English Europe	97293	10.7	109196	10.8	124192	10.5	129569	10.2	0.69
North Africa	27788	3.1	31158	3.1	34244	2.9	42357	3.3	0.55
Asia	35551	3.9	41319	4.1	48853	4.1	60561	4.8	0.01**
Others	72386	8.0	87342	8.7	109081	9.3	131653	10.4	0.07
unknown	14195	1.6	11137	1.1	7119	0.6	5443	0.4	<0.001
**Quartiles of SEIFA**									
1st quartile (most disadvantaged)	220402	24.3	246661	24.5	298947	25.4	345431	27.2	Ref
2nd quartile	215525	23.8	247467	24.6	302472	25.7	339819	26.7	0.95
3rd quartile	235066	26.0	261686	26.0	302528	25.7	305146	24.0	0.03*
4th quartile (most advantaged)	228795	25.3	246680	24.5	267063	22.7	272788	21.5	0.005**
unknown	5642	0.6	4752	0.5	7573	0.6	8181	0.6	0.92
**Major principal diagnostic diseases**									
Renal failure	260607	28.8	316603	31.4	406303	34.5	465423	36.6	0.08
Malignancy†	34685	3.8	37154	3.7	40862	3.5	42813	3.4	0.08
Diabetes with chronic complication	5182	0.6	8199	0.8	10610	0.9	10219	0.8	0.07
Congestive heart failure	868	0.1	1392	0.1	2260	0.2	2513	0.2	<0.001**
Metastatic solid tumour	4168	0.5	4956	0.5	5245	0.4	5919	0.5	0.84
Anaemia: deficiency	3044	0.3	4171	0.4	5448	0.5	6078	0.5	0.01**
Peripheral vascular disease	3719	0.4	3829	0.4	4468	0.4	4397	0.3	0.16
Chronic pulmonary disease	1856	0.2	2425	0.2	3695	0.3	3915	0.3	0.05*
liver disease	2907	0.3	2907	0.3	2961	0.3	2641	0.2	<0.001**
AIDS/HIV	786	0.1	632	0.06	573	0.05	0.03%		0.10
**Local health district of hospital**									
Metropolitan	613908	67.8	664395	66.0	750769	63.7	804378	63.3	Ref
Rural & Regional NSW	291522	32.2	342851	34.0	427814	36.3	466987	36.7	0.09
**Peer hospital groups**									
Principal referral group	513828	56.7	551821	54.8	622349	52.8	678371	53.4	Ref
Ungrouped acute	32494	3.6	33180	3.3	35262	3.0	35259	2.8	0.78
Major metro- and non-metropolitan	213892	23.6	261529	26.0	329707	28.0	361612	28.4	0.07
District group 1	71597	7.9	79165	7.9	96499	8.2	105335	8.3	0.41
District group 2	73619	8.1	81551	8.1	94766	8.0	90788	7.1	0.73
**Major surgical procedure**									
CABG	3279	0.4	2751	0.3	2662	0.2	2420	0.2	Ref
AAA repair	939	0.1	1097	0.1	1179	0.1	1042	0.1	0.02*
Hip replacement	4223	0.5	4625	0.5	4854	0.4	5308	0.4	<0.001**
Knee replacement	6055	0.7	7133	0.7	8414	0.7	8202	0.7	<0.001
Cholecystectomy	12554	1.4	12410	1.2	13308	1.1	12712	1.0	0.004**
Other	878380	97.0	979230	97.2	1148166	97.4	1241681	97.7	<0.001**

Note: *P≤0.05; **P≤0.01; †including lymphoma and leukaemia.

Ref =  reference group; SEIFA =  Socio-Economic Indices For Areas; CABG =  Coronary Artery Bypass Graft; AAA repair =  Abdominal Aortic Aneurysm repair.

### Crude and Adjusted Rates of Complications, FTR and 30-day Mortality

**Figure 2:**
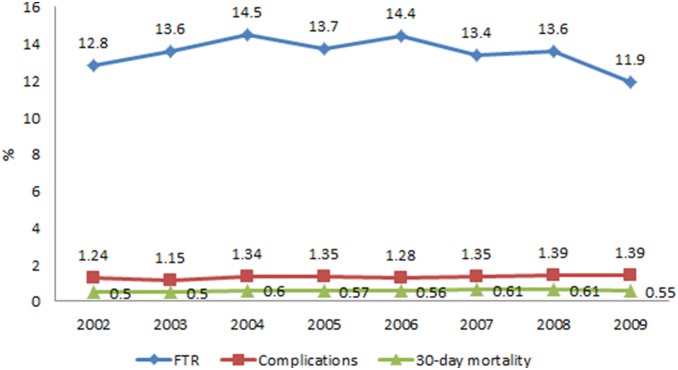
Adjusted rates of overall complications, failure-to-rescue, and 30-day mortality. Notes: P = 0.19 for complications; P =  (linear 0.012*; quadratic 0.005**) for failure-to-rescue; P = 0.28 for 30-day mortality.

Between 2002 and 2009, there were 60,092 surgical admissions (13.8 per 1,000 episodes) suffering at least one of the six complications after surgery, showing an unchanged trend across the study period (P = 0.19) (Table 2, Figure 2). During the same period, there were 8,446 (14.1%) deaths (FTR) related to those six complications during hospitalisation. The FTR rate showed a significant quadratic trend over the study period (P: linear = 0.012, quadratic = 0.005), increasing from 2002 to 2006 and then decreasing to 2009. The average incidence rate of 30-day mortality was 6.1 per 1,000 episodes, remaining stable over the study period (p = 0.28).

**Table 2:** Crude incidence rates and adjusted risk ratio (RR) of complications, failure-to-rescue and 30-day mortality.

Outcomes	2002	2003	2004	2005	2006	2007	2008	2009	Total
**No. of admissions**	449,955	455,475	480,646	526,600	568,742	609,841	623,256	648,109	4,362,624
**Complication**									
No. of cases	5,564	5,250	6,508	7,254	7,644	8,805	9,319	9,748	60,092
Incidence rate, per1,000 admissions‡	12.4	11.5	13.5	13.8	13.4	14.4	15.0	15.0	13.8
Adjusted RR(95%CI)	1.00	0.93(0.86–0.99)*	1.08(0.97–1.20)	1.09(0.95–1.25)	1.05(0.90–1.21)	1.10(0.95–1.28)	1.14(0.93–1.39)	1.13(0.93–1.38)	
**Failure-to-rescue**									
No. of deaths	710	710	948	1,043	1,168	1,250	1,370	1,247	8,446
Rate, %§	12.8	13.5	14.6	14.4	15.3	14.2	14.7	12.8	14.1
Adjusted RR(95%CI)	1.00	1.06(0.95–1.19)	1.13(1.00–1.27)*	1.12(0.96–1.30)	1.18(0.99–1.41)	1.10(0.92–1.30)	1.13(0.94–1.35)	0.99(0.84–1.15)	
**30-day mortality**									
No. of deaths	2,266	2,356	2,993	3,160	3,478	4,141	4,240	4,095	26,729
Incidence rate, per1,000 admissions†	5.0	5.2	6.2	6.0	6.1	6.8	6.8	6.3	6.1
Adjusted RR (95%CI)	1.00	1.00(0.91–1.11)	1.20(0.99–1.44)	1.15(0.92–1.45)	1.15(0.93–1.42)	1.25(1.02–1.55)*	1.25(0.93–1.69)	1.15(0.85–1.56)	

Note: 30-day mortality and complications were similar between the peer groups, but failure-to-rescue was less like to occur in the peer groups of major metro- and non-metropolitan (RR = 0.78; 95%CI: 0.60–0.99), district group 1 (RR = 0.57, 95%CI: 0.34–0.94) and district group 2 (RR = 0.61, 95%CI: 0.41–0.90) than that in the peer groups of principal referral and ungrouped acute hospitals.

‡ P = 0.053;

*P≤0.05;

§ P = (linear 0.02; quadratic 0.007);

† P = 0.12.

**Figure 3:**
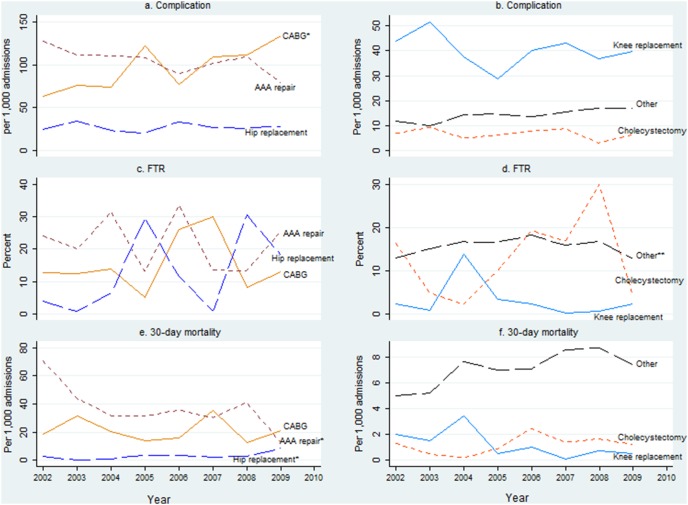
Adjusted rates of complications, FTR, and 30-day mortality, by major surgical procedure. Note: FTR  =  failure-to-rescue; CABG  =  Coronary Artery Bypass Graft; AAA repair  =  Abdominal Aortic Aneurysm repair; *(P = 0.03 for CABG in sub-figure a; P = 0.049 for AAA repair in sub-figure e; P = 0.041 for total hip replacement in sub-figure e). **P: (linear  = 0.012; quadratic = 0.005).

Adjusted complication rates over the eight-year period increased significantly for CABG only (P = 0.03) (Figure 3, a). Adjusted FTR rates fluctuated for CABG, AAA repair, total hip replacement, total knee replacement, cholecystectomy. However, the rates for the group of other surgery showed a quadratic trend with an initial increase between 2002 and 2006 followed by a decrease between 2006 and 2009 (P: linear = 0.012; quadratic = 0.005) (Figure 3, c and d). The adjusted rates of 30-day mortality significantly decreased for AAA repair (P = 0.049) and significantly increased for total hip replacement (P = 0.041). The rates were however, steady for other four groups during the same period (Figure 3, e and f).

### Variation of Patient Outcomes between and within Peer Hospital Groups

The incidence rates of all outcomes significantly varied between the best and worst quintile hospitals within each peer group (Table 3). There was a more than ten-fold difference in 30-day mortality incidence rates between best and worst quintile hospitals for district group 1 (2.0 vs 23.0 per 1,000 admissions; adjusted RR = 13.9, 95% CI: 4.27–45.49). Similarly, there was a more than six-fold difference in complication rates between the best and worst quintile hospitals for district group 2 (3.1 vs 18.8 per 1,000 admissions; adjusted RR = 6.4, 95% CI: 3.03–13.33). Compared to other hospital groups, district group 2 had the largest observed difference of FTR between the best and worst quintile hospitals (1.7 vs 21.8 per 1,000 admissions; adjusted RR = 8.1, 95% CI: 4.45–14.72). Principal referral hospitals had the smallest incidence ranges for all outcomes with a≤2.5-fold difference between the bottom and best quintiles. The variations in the rates of complications and 30-day mortality across peer groups were not statistically significant. However, compared to the principal referral hospital group, smaller hospitals, such as major metropolitan and non-metropolitan (RR = 0.78, 95% CI: 0.60–0.99), district group 1 (RR = 0.57, 95% CI: 0.34–0.94) and district group 2 (RR = 0.61, 95% CI 0.41–0.90) had a lower FTR rate.

**Table 3:** Crude incidence rates of complications, failure-to-rescue and 30-day mortality between the ‘best’ (top quintile) and ‘worst’ (bottom quintile) peer hospital group and adjusted risk ratio (RR) for the worst versus best, from 2002–2009.

	Complications	Failure-to-rescue	30-day mortality
	(per 1000 admissions)	(%)	(per 1000 admissions)
**Principal referral (n = 14)**			
Average	13	15.5	5.2
Best	9.5	8.7	2.8
Worst	17.7	21.2	7.8
Adjusted RR (95% CI)	1.8 (1.38–2.35)**	2.3 (1.90–2.88)**	2.5 (1.57–3.98)**
**Major metro- and non-metropolitan (n = 22)**			
Average	16.9	13	7
Best	8.1	7.1	2.2
Worst	31.1	18.1	13.4
Adjusted RR (95% CI)	5.3 (3.30–8.52)**	2.5 (1.89–3.41)**	8.4 (4.38–16.16)**
**District group 1 (n = 13)**			
Average	12.2	9.2	6.2
Best	5.9	4	2
Worst	27.3	18	23
Adjusted RR (95% CI)	5.4 (5.20–5.61)**	6.0 (3.47–10.47)**	13.9 (4.27–45.49)**
**District group 2 (n = 30)**			
Average	10	10.2	5.6
Best	3.1	1.7	1.5
Worst	18.8	21.8	13.7
Adjusted RR (95% CI)	6.4 (3.03–13.33)**	8.1 (4.45–14.72)**	7.1 (4.77–10.44)**

Note: **P≤0.01. We excluded ungrouped acute hospitals (A3) as there were only three hospitals and they were extremely different hospital types.

There were also significant variations in rates of complications, FTR, and 30-day mortality between the worst and best quintiles for each of the six surgical procedures (Table 4). The adjusted RR ranged from 1.9 to 3.4 for complications, from 2.2 to +∞ for FTR, and from 2.4 to +∞ for 30-day mortality. AAA repair surgery resulted in the highest rates in all three outcomes: complications (114.7 per 1,000 admissions), FTR (23.1%), and 30-day mortality (47.7 per 1,000 admissions). The largest variations in FTR and 30-day mortality emerged for three common procedures (i.e. hip replacement: 0 vs 15.8% and 0 vs 19.7 per 1,000 admissions; knee replacement: 0 vs 17.2% and 0 vs 9.6 per 1,000 admissions; and, cholecystectomy: 0 vs 42.3% and 0.3 vs 5.2 per 1,000 admissions). On average, patients undergoing cholecystectomy had the lowest rate of complications (8.9 per 1,000 admissions), but had the second highest rate of FTR (17.7%). Total knee replacement surgery resulted in the lowest rates of both FTR (1.9%) and 30-day mortality (1.5 per 1000 admissions), with a complication rate ranked in the middle of the six groups of procedures.

**Table 4:** Crude incidence rates of complications, failure-to-rescue and 30-day mortality for the ‘best’ (top quintile) and ‘worst’ (bottom quintile) principal referral hospitals and adjusted risk ratio (RR) for the worst versus best, from 2002–2009.

Type of procedure	Complications (per 1,000 admission)				Failure-to-rescue (%)				30-day mortality (per 1,000 admission)			
	Adjusted RR				Adjusted RR				Adjusted RR			
	Average	Best	Worst	(95% CI)	Average	Best	Worst	(95% CI)	Average	Best	Worst	(95% CI)
CABG (n = 11,111)	77.2	55.5	99.2	1.9 (1.75–2.03)**	13.8	5.4	23.1	6.3 (3.55–11.34)**	19.9	11.2	26.7	2.4 (1.49–3.99)*
AAA repair (n = 3,626)	114.7	76.6	234.6	3.4 (1.87–1.14)**	23.1	9.6	34.1	3.7 (2.28–6.02)**	47.7	25.0	90.2	3.9 (2.60–5.96)**
Hip replacement (n = 8.959)	27.6	16.3	53.6	3.2 (3.05–3.34))**	6.1	0	15.8	+∞	2.9	0	19.7	+∞
Knee replacement (n = 13,383)	47.2	26.4	85.1	2.5 (2.03–3.05)**	1.9	0	17.2	+∞	1.5	0	9.6	+∞
Cholecystectomy (n = 15,799)	8.9	4.6	16.9	2.7 (1.64–4.30)**	17.7	0	42.3	+∞	2.0	0.3	5.2	19.1 (8.00–45.81)**
Other (n = 2,313,491)	12.3	8.7	16.7	2.5 (2.04–3.02)**	15.8	9.3	21.2	2.2 (1.81–2.71)**	5.2	2.8	7.8	2.6 (1.60–4.10)**

Note: *P≤0.05;

**P≤0.01. CABG = Coronary Artery Bypass Graft; AAA repair = Abdominal Aortic Aneurysm repair.

## Discussion

We conducted a population-based study investigating trends and variations in the rates of surgical complications, FTR and 30-day mortality among all public acute hospitals in NSW, Australia over an 8-year period. We found that approximately 14 per 1000 hospital elective surgical admissions experienced at least one of six complications showing a stable incidence rate occurrence between 2002 and 2009. On average, around 14% of patients who had one of the six complications died during their hospital stay, accounting for 38% of all surgical hospital deaths. The rate of FTR increased between 2002 and 2006 then decreased afterwards. The rate of 30-day mortality was 6 per 1000 admissions and relatively stable between 2002 and 2009. Moreover, we found significant variations in the difference in the rates of complications, FTR and 30-day mortality between the best and worst quintile hospitals within each peer hospital group. The principal referral hospitals had a significantly higher overall rate of FTR in comparison to other peer hospital groups. We also found that there were modest variations in complication rates but marked significant differences in the rates of FTR and 30-day mortality across the six groups of surgical procedures between the best and the worst hospitals in the principal referral group.

To date, our study is the first in Australia to investigate trends and variations in the rates of surgical complications, FTR and 30-day mortality across all NSW public acute hospitals. The most recently published US benchmark data [30] reported an overall FTR rate of 11.9%, with a range of 6% for patients in the 18–39 years group and 12.1% in the 75+ years group, using the 2010 Healthcare Cost and Utilisation Project (HCUP) data. A recent large study [31] in the United Kingdom (UK) using the National Health Services (NHS) Commissioning Data Sets (CDS; 1997–2009), which included 146 hospital trusts and all (over 66 million) surgical admissions, reported a 9.1% FTR and 3.8% complication rate. Moreover, the UK study reported that the FTR rate was relatively stable during the study period but the complication rate increased from 3.6% in 1997–1998 to 4.2% in 2008–2009; three times as high as the complication rate reported in our study (1.4%). The UK study also reports that patients aged 75+ years had a 20.6% FTR rate. Another recent study [32] in the US found that the complication rate was much higher in patients aged 65+ years, accounting for 32.7–36.4% of all complications. The subsequent death rates after the complications varied from 6.8–16.7% between the best and worst hospitals.

It should be noted that reported rates of complications, FTR and 30-day mortality from individual centres are highly dependent on the complication definition adopted [33], [34], patient characteristics and co-morbidities [33], [35], surgical sub-specialties [36], [37], and features of the hospital [38], [39]. For example, a 2002 Australian teaching hospital study found that 16.9% of surgical patients suffered from at least one adverse event (very broadly defined complications), and 7% of them died as a result of an adverse event during their hospital stay [5].

Our study was consistent with those previously reported [32], [40], finding a large variability in FTR rates between the best and worst performing hospitals. However, our results indicate that large volume hospitals (i.e. principal referral) had a higher FTR rate compared to other peer hospital groups (low volume hospitals). This is in contrast to the reports of an association between high patient volume and better FTR outcome [41]–[43], but supports the findings that the volume-outcome relationship may be confounded by many other factors such as the level of nurse staffing [44], patient insurance status [45], severity of illness [33], level of ICU [46], and overall staffing level of the hospital [47].

Despite the relatively stable rates of complications and 30-day hospital mortality, the findings that FTR was increasing until 2006 and decreased afterwards has significant policy relevance. In Australia, patient safety agencies such as the National Australian Commission on Quality and Safety in Health Care and state-based Clinical Excellence Commission (CEC) of NSW were founded in 2006 and August 2004, respectively. Both agencies launched a wide range of patient safety programs targeting the quality of health care. For example, the Collaborating Hospitals’ Audit of Surgical Mortality (CHASM) which was implemented as a systematic peer-reviewed audit of surgically-related deaths in NSW and was reported as being beneficial to surgical practice improvement and the reduction of deaths [48]. Other programs initiated in NSW by the CEC, such as ‘Prevention of hospital-acquired infections’, ‘Sepsis Kills campaign’, ‘Quality indicator monitoring and public reporting’, ‘Clinical improvement and leadership program’, and ‘Towards a safer culture’, may have also contributed to the changes in FTR rates seen in this study.

The increasing complication rates for CABG in our study were possibly, in part, attributed to the fact that patients undergoing CABG were getting older with more comorbidities. The recent decrease in FTR rates among all surgical patients was especially encouraging considering that older patients are associated with an increased risk of FTR [5], [49] and that the average patient age undergoing elective surgery had increased over the past decade. It is worth noting that the five individual surgical procedure groups (i.e. CABG, AAA repair, total hip replacement, total knee replacement, Cholecystectomy) did not exhibit the similar clear trends of FTR possibly due to the relatively small number of operations (less than 3% of total surgical procedures done). However, all other surgical procedures combined showed the same quadratic trend.

In our study, the rates of complications, FTR and 30-day mortality varied markedly between the best and worst quintile hospitals in every peer hospital group, notably in district groups 1 and 2. The smallest variations in all three outcomes between best and worst quintile occurred in principal referral hospitals, suggesting fewer quality differences in those hospitals. The very large variations in the three outcomes between best and worst quintile of the district hospital groups suggests a greater need for improvement of post-surgical care in smaller sized hospitals. However, our study results also suggest that even within principal referral hospitals, there were still marked variations in rates of complications, FTR and 30-day mortality for the same surgical procedure. In particular, such variations were more striking for rates of FTR and 30-day mortality between the best and worst quintile hospitals, suggesting that many patient lives could be potentially saved should such variation be reduced in some degree. For example, the adjusted RR of FTR rates for worst versus best quintile were greater in our study compared with the variations for the same surgery in the US (adjusted RR: 6.8 in our study vs 3.1 in US study for CABG; 3.7 in our study vs 1.6 in US study for AAA repair) [32], [50]. The variations in 30-day mortality for high-risk surgical procedures in our study were much higher than that reported in the US (adjusted RR: 2.4 in our study vs 1.9 in US study for CABG; 3.9 in our study vs 2.5 in US study for AAA repair [50], [51]. Moreover, the variations were greater for FTR than that for 30-day mortality, suggesting that further attention to major surgical performance in relation to patient safety using FTR as an indicator may be warranted in NSW.

Principal referral hospitals had a higher rate of FTR compared to other peer hospital groups in our study, in contrast to previous reports that large teaching hospitals provide better quality surgical care and have lower mortality [42], [43], [52], [53]. One possible reason for this discrepancy could be that the case-mix, complexity and severity of surgical patients in principal referral hospitals in NSW are different from that of patients in other hospital groups (it is common practice for other peer hospital groups to transfer complex patients to principal referral hospitals). In addition, the differences in case-mix may, to a lesser extent, contribute to the differences between the best and worst hospitals within each peer hospital group. This suggests that government agencies such as the Bureau of Health Information of NSW and the National Health Performance Authority should consider reporting the post-surgical rates of complications, FTR and 30-day mortality, benchmarked within each peer hospital group with an appropriately tested risk-adjustment strategy. Moreover, after particular high-risk surgical procedures these rates may also be beneficial towards avoiding the potential difficulties introduced by differences in case-mix between different peer hospital groups.

Our findings that the rates of complications and 30-day mortality were similar but FTR rates varied between different peer hospital groups suggest that the FTR rate may not be closely associated with the overall mortality rate. The FTR rate reflects the ability of a hospital to detect and provide appropriate care to surgical patients after a complication has occurred. However, the rate of complications can also be an important indicator of the quality of care leading up to the complication(s). Despite the fact that we analysed all six complications together, each may have specific implications reflecting different aspects of care. For example, the degree of success in controlling hospital-acquired infection may, in part, contribute to the rate of sepsis. The high rate of cardiac arrest could be due to the lack of an early detection of deteriorating patients and rapid response system [54]. Similarly, whilst a death after cardiac arrest is an indicator of failure-of-rescue, the incidence rate of cardiac arrest in the first place is an indicator of failure to prevent. Thus, the higher rate of sepsis or cardiac arrest in a hospital may also reflect its preceding quality of care, contradicting the arguments that complications were mostly decided by patient characteristics rather than quality of care [1], [34]. A recent study in the US [55] showed that despite the rate of FTR decreased over the study years, in five out of the seven years there was a significant increase in patient safety indicators directly associated with surgery: accidental puncture/laceration, post-operative physiologic and metabolic derangement, post-operative sepsis, post-operative pulmonary embolus or deep vein thrombosis, and post-operative respiratory failure. The US authors discussed the possibility that the reduced FTR could be due to the increased use of Do Not Resuscitate (DNR) orders upon admission, leading to a better selection of candidates for resuscitation, better training of code teams, more rested resident physicians, and perhaps the implementation of rapid response teams [56] Therefore, it is important that the rates of complications and 30-day mortality should also be reported when considering FTR rates.

Our study was the first population-based study of all public acute hospitals, in the largest health jurisdiction in Australia, to present the change in the rates of complications, FTR and 30-day mortality among elective surgical patients. It adds weight to findings from similar studies in the US and Europe [55], [57], [58]. The current study used administrative data based on the ICD-10-AM which were extracted by certified professional coders based on standardised guidelines at each hospital, minimising potential investigator biases. The analyses and methodology can be repeated in the future at both a state and national level, offering a means for tracking and benchmarking the performance of surgical care among hospitals. Our study also had several limitations. Firstly, this was an observational study and as a result, no causality should be assumed among the relationships identified. Secondly, our study did not explore changes in the pattern of complication-specific postoperative mortality rates. Future analyses may shed more light if each component of the complication such as renal failure, sepsis, and shock/cardiac arrest is analysed separately. Thirdly, despite the evidence that FTR is a robust measure in showing a link between the key determinants of quality of surgical care (such as nurse-patient ratio, nurse work environment, hospital technology, hospital size and surgical volume level) and patients outcomes, the sensitivity and specificity of FTR may still be improved through the Present at Admission (POA) coding which wasunavailable for the current study [2], [59].

## Conclusion

Despite the relatively stable trends in the rates of complications and 30-day mortality over the study period, the rate of FTR has decreased since 2006 after an initial upward trend since 2002, possibly due to the wide range of patient safety programs introduced in NSW by peak patient safety agencies. The marked variations in the three rates between the best and worst quintile hospitals (among all peer hospital groups) highlight the opportunity for further quality improvement. The significant difference in FTR between each group of hospitals warrants further investigation. The potential of the three rates as a whole, among other indicators, for use as surgical performance indicators and a screening tool across Australia could be further explored. Further research is needed to investigate the implications of different ways for defining and identifying complications, and reporting complication- and procedure-specific postoperative mortality.

## Supporting Information

Appendix S1 Procedure codes from ICD-10-AM for selected surgical procedures.
(DOCX)
